# Characterization of respiratory bacterial co-infection and assessment of empirical antibiotic treatment in patients with COVID-19 at hospital admission

**DOI:** 10.1038/s41598-023-46692-x

**Published:** 2023-11-07

**Authors:** Adrián Antuori, Montserrat Giménez, Georgina Linares, Pere-Joan Cardona

**Affiliations:** 1 Microbiology Department, Clinical Laboratory North Metropolitan Area, Germans Trias i Pujol University Hospital, 08916 Badalona, Spain; 2 Centro de Investigación Biomédica en Red de Enfermedades Respiratorias (CIBERES), 28029 Madrid, Spain; 3 Genetics and Microbiology Department, Universitat Autònoma de Barcelona, 08913 Cerdanyola del Vallès, Spain

**Keywords:** Risk factors, Infectious diseases, Antimicrobials, Bacteria, Clinical microbiology

## Abstract

Accurate characterization of respiratory bacterial co-infection is critical for guiding empirical antibiotic treatment for hospitalised patients with coronavirus disease 2019 (COVID-19). We retrospectively assessed the clinical and analytical predictors of respiratory bacterial co-infection and described the empirical use of antibiotics in COVID-19 hospitalised patients. Respiratory bacterial co-infection was documented in 6.9% (80/1157) of the patients. The predominant bacteria isolates were *Haemophilus influenzae*, followed by *Streptococcus pneumoniae* and *Pseudomonas aeruginosa*. Respiratory bacterial co-infection was associated with having had a positive culture for a respiratory pathogen in the last year (OR = 25.89), dyslipidaemia (OR = 2.52), heart failure (OR = 7.68), ferritin levels < 402 ng/mL (OR = 2.28), leukocyte count > 8.7 × 10^9^/L (OR = 2.4), and patients with chronic obstructive pulmonary disease treated with inhaled corticosteroids (OR = 12.94). Empirical antibiotic treatment was administered in 42.33% of patients, although it declined across the distinct study periods (*p* < 0.001). Patients admitted to intensive care units harbouring co-infection exhibited worse outcomes and more bacterial secondary infections. In conclusion, respiratory bacterial co-infection prevalence was low, although it could lead to unfavourable outcomes. Moreover, the percentage of empirical antibiotic treatment remained high. The study's findings allowed the identification of several predictors for respiratory bacterial co-infection and could help implement adequate antibiotic stewardship measures.

## Introduction

The identification of bacterial co-infection in hospitalised patients with coronavirus disease 2019 (COVID-19) has posed a challenge since the pandemic's inception. Early-stage studies and meta-analyses have consistently documented low bacterial co-infection rates upon patient admission^1–3^, although some studies have associated bacterial co-infection with adverse outcomes -such as heightened mortality, the need for mechanical ventilation, and prolonged hospital stays-^3, 4^. However, data on predisposing risk factors for bacterial co-infection remain scarce.

Recent meta-analysis data revealed antibiotic usage prevalence exceeding 70.0% among COVID-19 patients^2^. In the first months of the pandemic, there was extensive empirical use of azithromycin because its antiviral effect and immunomodulatory properties^5^. Maintaining vigilance over bacterial co-infection dynamics is essential for steering appropriate empirical antibiotic treatments in hospitalised COVID-19 patients.

To accomplish this, we conducted an analysis on COVID-19 patients admitted at a tertiary hospital between March 2020 and April 2021 to assess the clinical and analytical predictors of respiratory bacterial co-infection. We have also described the empirical antibiotic treatment admission during different periods of the COVID-19 pandemic and evaluated the impact of antibiotic use in patients admitted to intensive care units (ICUs).

## Methods

### Study design

We performed an observational retrospective study at the Germans Trias i Pujol University Hospital (Badalona, Spain), the designated referral hospital of an urban area with 1,400,000 inhabitants. We have included all patients ≥ 18 years old with a diagnosis of COVID-19 (positive nucleic acid amplification test (NAAT) for SARS-CoV-2 on a nasopharyngeal swab or respiratory tract secretions), and admitted for > 48 h between March 2020 and April 2021. To be eligible for the study, patients should have a positive SARS-CoV-2 NAAT result within three days preceding or two days subsequent admission.

For the analysis of predictors related to respiratory bacterial co-infection, patients were included if they had undergone collection of at least one urine sample for *Legionella pneumophila* and *Streptococcus pneumoniae* antigens (Sofia-Quidel, San Diego, USA) within the initial 48 h following admission. Patients were excluded from the analysis if any indications of bacterial co-infection originating from sources other than respiratory infection were detected (refer to the Definitions section).

### Data collection

Demographic information, medical history, comorbid conditions, laboratory test, microbiological data, treatment, ICU admission, and outcomes were obtained electronically from an anonymised database known as *CORE-COVID*, implemented at the hospital. This comprehensive repository aggregates data from eight hospitals and primary healthcare systems, encompassing patients admitted with confirmed COVID-19 diagnoses.

### Microbiological procedures and definitions

We performed standard microbiological procedures for the investigation of bacterial and fungal pathogens. *S. pneumoniae*, *L. pneumophila*, *Haemophilus influenzae*, *Moraxella catarrhalis*, *Staphylococcus aureus*, *Pseudomonas aeruginosa*, *Achromobacter xylosoxidans*, *Stenotrophomas maltophila*, *Burkholderia spp*, *Aspergillus spp*, asa well as microorganisms within the *Enterobacterales order* and *Mycobacteriaceae* family were considered as respiratory pathogens.

Respiratory bacterial co-infection was defined as the growth of respiratory pathogens obtained from blood, pleural fluids, good-quality sputum and endotracheal aspirates (> 25 polymorphonuclear leukocytes and < 25 epithelial cells), bronchoalveolar lavages and positive urinary antigen test for *S. pneumoniae* or *L. pneumophila* (Sofia-Quidel, San Diego, USA) within the initial 48 h following admission. As mentioned above, patients demonstrating evidence of bacterial or fungal co-infection from sources other than respiratory infection were excluded from the study.

We characterized secondary infections as any positive urine, respiratory tract, or blood cultures emerging 48 h post-admission. We also recorded the proportion of secondary infections attributable to extended-spectrum beta lactamase (ESBL) microorganisms, methicillin-resistant *S. aureus* (MRSA), vancomycin-resistant enterococci (VRE), and extensively drug-resistant *P. aeruginosa* were recorded^6^. These three categories were considered multidrug-resistant infections. Additionally, we documented secondary infections involving *Clostridioides difficile*. Days of Therapy (DOT) was calculated for each patient and standardised for 100 patient days.

### Statistical analysis

First, we compared patients with and without respiratory bacterial co-infection and performed a univariate and multivariate logistic regression model to explore the predictors associated with respiratory bacterial co-infections. Variables with a significance level of < 0.10 in the univariate analysis were included in the multivariate analysis. Odds ratios (ORs) with their 95% CI values were calculated.

Second, we performed a descriptive analysis of empirical antibiotic in hospitalised COVID-19 patients, categorizing them into four distinct study periods aligned with Spain's first four COVID-19 waves (1st: from March 2020 to May 2020; 2nd: from June 2020 to December 9, 2020; 3rd: from December 10 to March 15, 2021, and 4th: March 16, 2021, to May 1, 2021).

Third, we compared patients admitted to ICUs with and without antibiotic therapy within 48 h of admission to evaluate the impact of empirical antibiotic use within a homogeneous population.

We determined continuous and categorical variables as median (interquartile range (IQR)) and absolute number (percentage), respectively. The Mann–Whitney U, chi-square, and Fisher´s exact tests were performed for descriptive analysis. Significance was set at *p* < 0.05. We performed the statistical studies using the software SPSS Statistics 24.0 (IBM, Armonk, NY, USA).

### Ethics approval

The study was conducted according to the principles of the Declaration of Helsinki and was approved by the Ethics Committee of the Germans Trias i Pujol University Hospital (approval application number PI-21-073). The Ethics Committee of the Germans Trias i Pujol University Hospital waived the requirement for informed consent because of the study's retrospective nature, and all data were analysed anonymously. All methods of the study were carried out in accordance with institutional guidelines and regulations.

## Results

### Participant characteristics and respiratory bacterial co-infections

Of the 4858 people in the *CORE-COVID* database, 2121 (43.66%) were eligible for the study. Within this subset, 1157 (54.55%) underwent assessment for predictors of respiratory bacterial co-infection. Respiratory bacterial co-infection was documented in 6.9% (80/1157) of the patients (6.2% in non-ICU patients and 8.9% in ICU patients; *p* = 0.001). 3.5% of the patients (41/1157) had a positive urinary antigen test for *S. pneumoniae,* while one yielded a positive urinary test for *L. pneumophila*. Furthermore, 3.8% (44/1157) presented a positive bacterial culture from respiratory tract specimens. A microorganism was isolated in blood cultures in 0.2% of cases (3/1157), two attributed to *S. pneumoniae* and one to *S. aureus*. The most prevalent bacteria isolated from respiratory tract specimens were *H. influenzae* (16.3%, 8/49), followed by *S. pneumoniae* (14.3%, 7/49)*, P. aeruginosa* (14.3%, 7/49), *S. aureus* (14.3%, 7/49), and *Escherichia coli* (10.2%, 5/49). Five (11.4%, 5/49) mixed infections were detected (caused by *Achromobacter xylosoxidans*/*Acinetobacter spp.*, *S. pneumoniae*/*Enterobacter cloacae*, *E. coli*/*S. aureus,* and *Stenotrophomonas maltophilia*/*E. coli*).

Main demographic information, patient characteristics, laboratory findings, treatments, and outcomes by respiratory groups are shown in Table 1. Patients with co-infection exhibited greater requirements for invasive mechanical ventilation (28.8% *vs* 19.0%, *p* = 0.035), prolonged hospital stays (14 *vs* 11 days, *p* = 0.003), elevated 30-day readmission rates (10.0% *vs* 4.2%, *p* = 0.025), and a higher prevalence of respiratory (23.8% *vs* 11.2%, *p* = 0.001) and urinary bacterial secondary infections (21.3% *vs* 10.6%, *p* = 0.004) (Supplementary table 1).

**Table 1:** Main epidemiological and clinical characteristics of hospitalized COVID-19 patients.

	NO co-infection (*n* = 1077)	Respiratory co-infection (*n* = 80)	Total (*N* = 1157)
Socio-demographic characteristics			
Age	63.0 (53.0–74.0)	68.5 (57.0–77.0)	65.0 (53.0–76.0)
Male gender	659 (61.2)	46 (57.5)	705 (60.9)
Medical history			
Positive culture for a respiratory pathogen in the last year	9 (0.8)	10 (12.5)	19 (1.6)
Treatment before admission			
Inhaled corticosteroids	47 (4.4)	14 (17.5)	61 (5.3)
Oral corticosteroids	18 (1.7)	4 (5.0)	22 (1.9)
Treatment at admission^+^			
Antibiotic	609 (56.5)	44 (55.0)	653 (56.4)
Comorbidities			
Hypertension	460 (42.7)	40 (50.0)	500 (43.2)
Dyslipidemia	554 (51.4)	53 (66.3)	607 (52.5)
Diabetes mellitus 2	309 (28.7)	32 (40.0)	341 (29.5)
Body mass index > 35	178 (31.4)	9 (24.3)	187 (31.0)
Chronic kidney failure	179 (16.6)	21 (26.3)	200 (17.3)
Heart failure	65 (6.0)	12 (15.0)	77 (6.7)
Chronic obstructive pulmonary disease	119 (11.0)	23 (28.8)	142 (12.3)
HIV	9 (0.8)	0 (0.0)	9 (0.8)
Solid tumor	88 (8.2)	4 (5.0)	92 (8.0)
Hematological malignancy	25 (2.3)	1 (1.3)	26 (2.2)
Solid organ transplantation	16 (1.5)	4 (5.0)	20 (1.7)
Cirrhosis	10 (0.9)	1 (1.3)	11 (1.0)
Vital signs at admission^+^			
Temperature (°C)	36.6 (35.9–37.1)	36.4 (35.8–37.6)	36.5 (35.9–36.9)
APACHE	16.0 (12.0–22.5)	15.0 (13.3–23.8)	16.0 (13.0–23.3)
SOFA	2.53 (1.0–3.0)	2.73 (2.0–4)	2.56 (1.0–3.25)
Laboratory parameters at admission^+^			
Ferritin (ng/mL)	458.0 (302.5–1132.5)	402.0 (160.0–883.0)	494.0 (243.0–1002.0)
Leukocyte count (× 10^9^/L)	6.7 (5.0–9.3)	8.7 (6.3–13.00)	6.6 (5.0–9.2)
Neutrophil count (× 10^9^/L)	5.1 (3.5–7.7)	6.8 (4.8–11.1)	4.9 (3.5–7.4)
C-RP (mg/L)	97.7 (51.8–167.0)	101.20 (50.6–170.9)	80.3 (33.8–142.5)
Procalcitonin (ng/mL)^**+**^	0.12 (0.06–0.34)	0.2 (0.06–0.78)	0.1 (0.05–0.27)
Secondary infections*			
Bacteremia	52 (4.8)	6 (7.5)	58 (5.0)
Respiratory bacterial infection	121 (11.2)	19 (23.8)	140 (12.1)
Urinary tract infection	114 (10.6)	17 (21.3)	131 (11.3)
*Clostridioides difficile*	3 (0.3)	1 (1.3)	4 (0.3)
ESBL**	23 (12.6)	4 (14.8)	27 (12.9)
MRSA**	4 (3.8)	1 (3.7)	5 (3.8)
Outcomes			
ICU admission	264 (24.5)	26 (32.5)	290 (25.1)
Invasive mechanical ventilation*	205 (19.0)	23 (28.8)	228 (19.7)
ECMO*	78 (7.2)	13 (16.3)	91 (7.9)
Length of hospital stay (days)	11 (6–18)	14 (7.3–32)	10 (6–17)
30-day readmissions	45 (4.2)	8 (10.0)	53 (4.6)
Death	198 (18.4)	16 (20.0)	214 (18.6)

*MRSA* Methicillin-Resistance Staphylococcus aureus; *ESBL* Extended-spectrum β-lactamases; *ECMO* Extracorporeal Membrane Oxygenation.

^**+**^Within the first 48 h after admission; *After 48 h of admission; **Percentage of total bacteremia, respiratory bacterial infection, and urinary tract infection.

### Predictors of respiratory bacterial co-infection

**Figure 1:**
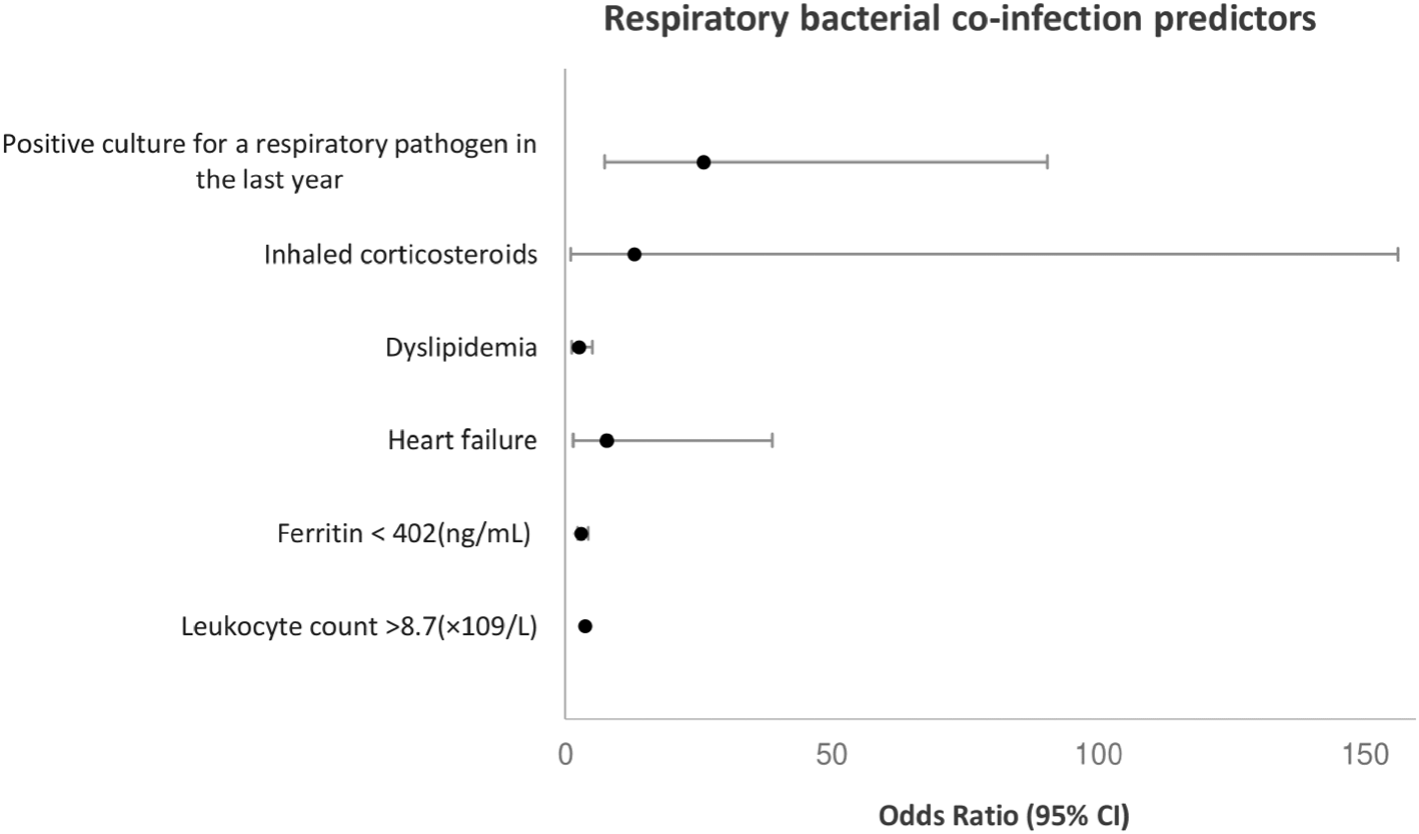
Predictors of respiratory bacterial co-infection by multivariate logistic regression analyses. Culture for a respiratory pathogen in the last year (*p* < 0.001, OR = 25.89; 95% CI, 7.40–90.49), dyslipidemia (*p* = 0.010, OR = 2.52; 95% CI, 1.25–5.08), heart failure (*p* = 0.015, OR = 7.68; 95% CI, 1.48–38.90), ferritin levels < 402 ng/mL (*p* = 0.011, OR = 2.28; 95% CI, 1.21–4.29), leukocyte count > 8.7 × 10^9^/L (*p* = 0.004, OR = 2.40; 95% CI, 1.26–4.45), and patients with chronic obstructive pulmonary disease (COPD) treated with inhaled corticosteroids (ICS) (*p* = 0.044, OR = 12.94; 95% CI, 1.07–156.30).

In the adjusted analyses (Fig. 1), respiratory bacterial co-infection was associated with having a positive culture for a respiratory pathogen in the last year (*p* < 0.001, OR = 25.89; 95% CI, 7.40–90.49), dyslipidaemia (*p* = 0.010, OR = 2.52; 95% CI, 1.25–5.08), heart failure (*p* = 0.015, OR = 7.68; 95% CI, 1.48–38.90), ferritin levels < 402 ng/mL (*p* = 0.011, OR = 2.28; 95% CI, 1.21–4.29), leukocyte count > 8.7 × 10^9^/L (*p* = 0.004, OR = 2.40; 95% CI, 1.26–4.45), and patients with chronic obstructive pulmonary disease (COPD) treated with inhaled corticosteroids (ICS) (*p* = 0.044, OR = 12.94; 95% CI, 1.07–156.30).

### Empirical antimicrobial treatment at admission

Overall, 42.33% (*n* = 898/2121 patients with a positive SARS-CoV-2 NAAT) of eligible patients were treated with antibiotics. Antibiotic treatment rates were similar between patients with and without a bacterial co-infection (55.0% *vs* 56.5%, *p* = 0.788).

**Figure 2:**
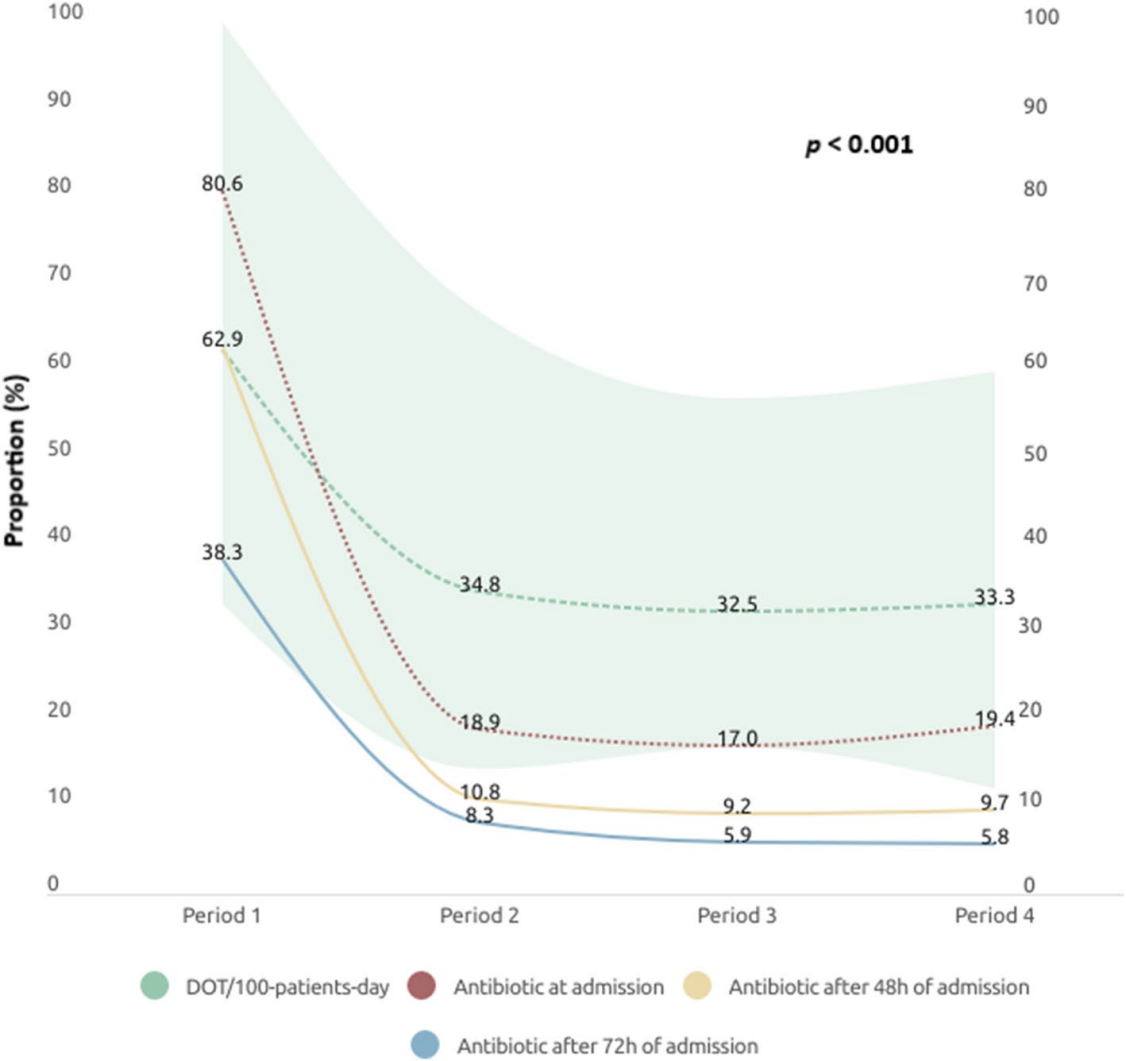
Proportion of antibiotic use in hospitalised patients with COVID at admission (red), 48 (yellow) and 72 h (blue) post-admission, and Days of Therapy (DOT) per 100 patient days across different study periods (period 1: from March 2020 to May 2020; period 2: from June 2020 to December 9, 2020; period 3: from December 10 to March 15, 2021, and period 4: March 16, 2021, to May 1, 2021).

Ceftriaxone (81.1%, 729/898) and azithromycin (74.7%, 671/898) were the antibiotics most commonly used. The proportion of patients with antibiotics at admission decreased significantly across the different study periods (*p* < 0.001), as did the proportion of patients whose treatment was discontinued at 48 (*p* < 0.001) and 72 h (*p* < 0.001) post-admission (Supplementary table 2). In addition, the treatment duration, measured in DOT per 100 patient days, displayed an extended duration in the first period compared to the subsequent periods (Fig. 2).

### Impact of antibiotic treatment in patients admitted to ICUs

We observed no differences in mortality rates (55.0% *vs* 42.0%, *p* = 0.519), length of hospital stay (24 *vs* 22 days, *p* = 0.339), and 30-day readmission rates (3.0% *vs* 5.0%, *p* = 0.278) among ICU patients who had received antibiotics compared those who had not (Supplementary table 3). In addition, secondary infection rates (bacteremia: 21.8% *vs* 16.7%, *p* = 0.222; respiratory bacterial infection: 42.3% *vs* 44.7%, *p* = 0.648; urinary tract infection: 29.5% *vs* 34.0, *p* = 0.365), and percentages of multidrug-resistant microorganisms (ESBL: 9.5% *vs* 8.6%, *p* = 0.667; MRSA: 0.9% *vs* 2.6%, *p* = 0.192, XDR *Pseudomonas aeruginosa*: 2.3% vs 2.0 *p* = 0.813) did not differ between the two groups. Finally, the consumption of antibiotics, measured in DOT per 100 patient days, was higher in patients who had received antibiotics at admission (38.3 vs 26.6 days, *p* = 0.005) (Supplementary table 3).

## Discussion

The current study provides a comprehensive characterization of respiratory bacterial co-infection, and the use and impact of antibiotics in clinical progression among hospitalised COVID-19 patients. Respiratory bacterial co-infection was documented in 6.9% of the patients, slightly higher than the percentage reported in a recent review, which described a co-infection rate of 4.4% among hospitalised patients^2, 3, 7^. This divergence could be attributed to the selection criteria of this study, in which patients were included if at least one urine sample for *L. pneumophila* and *S. pneumoniae* antigens was collected. In contrast, in other studies with lower co-infection rates, detection of *S. pneumoniae* antigen in urine was performed in fewer than 25% of patients^3, 7^. Conversely, a study conducted in Barcelona among hospitalized COVID-19 patients during the pandemic's initial year reported a co-infection rate approaching 10%. The authors hypothesised that this elevated percentage could stem from diagnostic tests being requested solely in cases of clinical suspicion. Another explanation revolves around the cessation of lockdowns after the pandemic's initial months, potentially facilitating increased community transmission of microorganisms like *S. pneumoniae*. Although the present study covers a similar period as the aforementioned study, most of the eligible patients in this study were from the pandemic's outset (72.7% within the initial two study periods), which may not reflect the changes mentioned by Moreno-Garcia et al*.*^8^. This underscores the significance of tracking co-infection rates in forthcoming years.

While typically, community-acquired pneumonia (CAP) etiological agents, such as *H. influenzae* (16.3%, 8/49) and *S. pneumoniae* (14.3%, 7/49), accounted for many confirmed respiratory bacterial co-infections, healthcare-related microorganisms, such as *P. aeruginosa* (14.3%), *S. aureus* (14.3%) or *E. coli* (10.2%), were not negligible. The high percentage of patients with comorbidities (approximately 30% with COPD), prone to respiratory tract infections by these microorganisms, might account for their detection in this study.

In agreement with other studies^3, 4^, patients with bacterial co-infections displayed unfavourable outcomes. They required more invasive mechanical ventilation, experienced prolonged hospital stays, exhibited increased 30-day readmission rates, and were prone to secondary respiratory and urinary bacterial infections. Surprisingly, only 55% of patients with a respiratory bacterial co-infection received antibiotic treatment. Despite the limitations of this observational study, there is a clear need to improve the identification of patients with bacterial co-infection.

We were able to describe the predictors of respiratory bacterial co-infection, which is essential to design specific treatment protocols. To the best of our knowledge, this is the first study to establish links between having had a positive culture for a respiratory pathogen in the last year (OR = 25.89), dyslipidaemia (OR = 2.52), heart failure (OR = 7.68), and patients with COPD treated with ICS (OR = 12.94), with respiratory bacterial co-infection. In addition, Ferritin levels < 402 ng/mL (OR = 2.28) and leukocyte count > 8.7 × 10^9^/L (OR = 2.4) were also associated with co-infection in adjusted analysis.

The presence of a positive culture for a respiratory pathogen in the preceding year exhibited a robust association with respiratory bacterial co-infection in this study. Previous works have also linked recent respiratory tract infections (caused by viruses and bacteria) to increased susceptibility for subsequent CAP^9, 10^.

Elevated leukocyte counts^11, 12^ and diminished ferritin levels^8^ have been previously associated with bacterial co-infection. Unlike other studies^8, 13^, we did not identify procalcitonin as an independent risk factor for bacterial co-infection. Although these biomarkers may not have sufficient sensitivity, specificity, or a positive predictive value by themselves, they can be combined with other clinical and radiological features to rule out bacterial co-infection with a high negative predictive value^13, 14^.

Dyslipidaemia has been linked to severe COVID-19 infections^15^ and higher rates of community-acquired sepsis^16^. Chronic inflammation and endothelial dysfunction^15^ may also facilitate bacterial co-infection in these patients. Moreover, as shown in the present study, chronic cardiovascular diseases, such as heart failure, have been described as a risk factor for CAP, and this association was to be expected^17^.

ICS are commonly employed to manage various chronic respiratory ailments like asthma or COPD, enhancing functionality, quality of life, and reducing exacerbation risks ^18^. However, ICS play a role in suppressing anti-bacterial host defences^19^ and increase the susceptibility to bacterial pneumonia^20^. This aspect could potentially favour the occurrence of bacterial co-infections among patients diagnosed with COVID-19.

As in other studies^1, 7^, the low percentage of patients with bacterial co-infection contrasts with the substantial number receiving antibiotics upon admission (42.33%). Nevertheless, the proportion of patients with antibiotics at admission decreased significantly in the different study periods, from 80.6% in the first study period (from March 2020 to May 2020) to 19.4% in the last study period (March 16, 2021, to May 1, 2021) (*p* < 0.001). This was also mirrored the patients whose treatment ceased at 48 and 72 h post-admission, probably due to the absence of data on bacterial co-infection, and the lack of local and international recommendations in the early stage of the pandemic. Furthermore, it was suggested that some antibiotics, such as azithromycin, could benefit in patients with COVID-19 due to their immunomodulatory properties, possibly driving over-treatment in these patients^21^.

Finally, we analysed the antibiotics use and their effects on a subset of ICU patients. We observed no differences in mortality, hospital stay duration, and 30-day readmission rates. Similarly, a study conducted in China found no association between early antibiotic use and mortality among non-severe COVID-19 patients admitted without bacterial infection. However, the patients treated with antibiotics were more at risk of progressing to severe illness and had protracted hospital stays. The authors suggested that antibiotic-induced dysbiosis might contribute to inflammation dysregulation in COVID-19 disease^22^.

Although we observed no differences in nosocomial infection rates and the prevalence of multidrug-resistant microorganisms (11.8% *vs* 14.0%, *p* = 0.616) between ICU patients who had received antibiotics at admission and those who had not, the DOT per 100 patient days was significantly greater in the first group (38.3 vs 26.6 days, *p* = 0.005). This could potentially wield significant consequences for the local ecology, potentially favouring the spread and outbreaks of high-risk clones^23^.

This study does exhibit some limitations. Despite encompassing an extensive study period (from March 2020 to May 2021), variations in bacterial co-infection rates and empirical antibiotic therapy may arise due to changes in clinical guidelines and the profile of patients admitted to the hospital, especially in terms of their severity. Secondly, the inclusion criteria involve patients who have been admitted for more than 48 h. This criteria excludes all patients who were discharged from the emergency department with antibiotic prescriptions, and it has the potential to favour the selection of patients with more severe clinical presentations. Thirdly, our definition of respiratory bacterial co-infection solely relies on retrospective positive cultures, rather than incorporating radiological or clinical features, potentially leading to both over and underdiagnosis. Nevertheless, the considerable number of patients in the study and the differences in septic and inflammatory parameters observed between the two groups strongly imply the presence of authentic bacterial co-infections. Fourthly, the previous use of antibiotics could have influenced the yield of bacterial cultures. However, we observed no significant differences between the co-infected (6.3%) and not co-infected groups (5.7%) in terms of the proportion of antibiotics administered prior to admission. Fifthly, we did not use non-culture tests, such as the nucleic acid amplification or serology assays, to detect respiratory pathogens. This could have potentially led to an underestimation of respiratory bacterial co-infection caused by pathogens such as *Chlamydia pneumoniae* and *Mycoplasma pneumoniae*^24^. However, a recent meta-analysis suggests that co-infections by these microorganisms were infrequent in COVID-19 patients^1^. Lastly, it's important to note that this study was conducted at a single centre, and the incidence and epidemiology of respiratory bacterial co-infections may differ across geographic areas.

In conclusion, respiratory bacterial co-infection was low in a large cohort of hospitalised COVID-19 patients, although it could lead to worse outcomes in these patients. In addition, the percentage of patients with empirical antibiotic treatment remained high, even though it declined from the initial phase of the pandemic. Furthermore, empirical antibiotic treatment did not show exhibit benefits in patients admitted to ICUs. The present study allows identifying clinical and analytical predictors of respiratory bacterial co-infection within this population and could thereby help design targeted diagnostic and empirical antibiotic treatment protocols. A more comprehensive understanding of respiratory bacterial co-infection is essential to effectively identify high-risk patients and to implement suitable antibiotic stewardship measures.

### Supplementary Information

Supplementary Tables.

## Data Availability

The study protocol, statistical analysis plan, and databases are available to anyone who requests them. These requests should be directed to Adrián Antuori (aantuori.germanstrias@gencat.cat).
