# Supplementary material for: Characterization of respiratory bacterial co-infection and assessment of empirical antibiotic treatment in patients with COVID-19 at hospital admission
Source: Sci Rep. 2023 Nov 7;13:19302. doi: 10.1038/s41598-023-46692-x (PMC10630415; doi:10.1038/s41598-023-46692-x)
Supplement: Supplementary file 1 — Supplementary Tables. [file 41598_2023_46692_MOESM1_ESM.doc]

**Characterization of respiratory bacterial co-infection and assessment of empirical antibiotic treatment in patients with COVID-19 at hospital admission**

Adrián Antuori^1^**^*^**, Montserrat Giménez^1^, Georgina Linares^1^, Pere-Joan Cardona^1,2,3^

1. Microbiology Department, North Metropolitan Clinical Laboratory, 'Germans Trias i Pujol' University Hospital, 08916 Badalona, Spain.

2. Centro de Investigación Biomédica en Red de Enfermedades Respiratorias (CIBERES), 28029 Madrid, Spain.

3. Genetics and Microbiology Department, Universitat Autònoma de Barcelona, 08913 Cerdanyola del Vallès, Spain.

**^*^Corresponding author**: Adrián Antuori. E-mail: [aantuori.germanstrias@gencat.cat](mailto:aantuori.germanstrias@gencat.cat). Telephone: 93 497 88 26

**Keywords:** SARS-CoV-2; COVID-19; bacterial co-infection; antibiotic; risk factors.

**Running title:** Bacterial co-infection in patients with COVID-19.

**Supplementary table 1. Univariate and multivariate logistic regression analyses of clinical and analytical predictors of respiratory bacterial co-infection.**

|  | **NO co-infection (*n* = 1077)** | **Respiratory co-infection**  **(*n* = 80)** | **Total (*N* = 1157)** | **Univariate model** | | | **Multivariate model** | | |
| --- | --- | --- | --- | --- | --- | --- | --- | --- | --- |
|  |  |  |  |  | ***p-value*** |  | **OR** | **95% CI** | ***p-value*** |
| **Socio-demographic characteristics** |  |  |  |  |  |  |  |  |  |
| Age | 63.0 (53.0-74.0) | 68.5 (57.0-77.0) | 65.0 (53.0-76.0) |  | **0.052** |  |  |  |  |
| Male gender | 659 (61.2) | 46 (57.5) | 705 (60.9) |  | 0.514 |  |  |  |  |
| **Medical history** |  |  |  |  |  |  |  |  |  |
| Hospitalized last year | 123 (11.4) | 21 (26.3) | 144 (12.4) |  | **<0.001** |  |  |  |  |
| Positive culture for a respiratory pathogen in the last year | 9 (0.8) | 10 (12.5) | 19 (1.6) |  | **<0.001** |  | 25.89 | 7.40-90.49 | **<0.001** |
| **Treatment before admission** |  |  |  |  |  |  |  |  |  |
| Immunomodulatory treatment | 5 (2.1) | 0 (0.0) | 5 (2.1) |  | 1.000 |  |  |  |  |
| Inhaled corticosteroids | 47 (4.4) | 14 (17.5) | 61 (5.3) |  | **<0.001** |  | 12.941 | 1.07- 156.30 | **0.044** |
| Oral corticosteroids | 18 (1.7) | 4 (5.0) | 22 (1.9) |  | 0.060 |  |  |  |  |
| **Treatment at admission^+^** |  |  |  |  |  |  |  |  |  |
| Steroids | 445 (41.3) | 46 (57.5) | 491 (42.4) |  | **0.005** |  |  |  |  |
| Antibiotic | 609 (56.5) | 44 (55.0) | 653 (56.4) |  | 0.788 |  |  |  |  |
| **Comorbidities** |  |  |  |  |  |  |  |  |  |
| Hypertension | 460 (42.7) | 40 (50.0) | 500 (43.2) |  | 0.204 |  |  |  |  |
| Dyslipidemia | 554 (51.4) | 53 (66.3) | 607 (52.5) |  | **0.010** |  | 2.52 | 1.25 - 5.08 | **0.010** |
| Smoking | 80 (7.4) | 9 (11.3) | 89 (7.7) |  | 0.216 |  |  |  |  |
| Alcoholism | 73 (6.8) | 12 (15.0) | 85 (7.3) |  | **0.007** |  |  |  |  |
| Diabetes mellitus 1 | 6 (0.6) | 0 (0.0) | 6 (0.5) |  | 1.000 |  |  |  |  |
| Diabetes mellitus 2 | 309 (28.7) | 32 (40.0) | 341 (29.5) |  | **0.041** |  |  |  |  |
| Body mass index > 35 | 178 (31.4) | 9 (24.3) | 187 (31.0) |  | 0.368 |  |  |  |  |
| Chronic kidney failure | 179 (16.6) | 21 (26.3) | 200 (17.3) |  | **0.028** |  |  |  |  |
| Hemodialysis | 7 (0.6) | 1 (1.3) | 8 (0.7) |  | 0.437 |  |  |  |  |
| Cerebrovascular disease | 65 (6.0) | 4 (5.0) | 69 (6.0) |  | 1.000 |  |  |  |  |
| Valvular heart disease | 75 (7.0) | 12 (15.0) | 87 (7.5) |  | **0.009** |  |  |  |  |
| Peripheral vasculopathy | 10 (0.9) | 2 (2.5) | 12 (1.0) |  | 0.199 |  |  |  |  |
| Ischemic cardiopathy | 200 (18.6) | 25 (31.3) | 225 (19.4) |  | **0.006** |  |  |  |  |
| Heart failure | 65 (6.0) | 12 (15.0) | 77 (6.7) |  | **0.002** |  | 7.68 | 1.48 - 38.90 | **0.015** |
| Sleep apnea/hypopnea syndrome | 120 (11.1) | 8 (10.0) | 128 (11.1) |  | 0.753 |  |  |  |  |
| Asthma | 91 (8.4) | 9 (11.3) | 100 (8.6) |  | 0.390 |  |  |  |  |
| Parkinson's | 20 (1.9) | 0 (0.0) | 20 (1.7) |  | 0.391 |  |  |  |  |
| Multiple sclerosis | 1 (0.1) | 0 (0.0) | 1 (0.1) |  | 1.000 |  |  |  |  |
| Chronic obstructive pulmonary disease | 119 (11.0) | 23 (28.8) | 142 (12.3) |  | **<0.001** |  |  |  |  |
| Rheumatoid arthritis | 13 (1.2) | 1 (1.3) | 14 (1.2) |  | 1.000 |  |  |  |  |
| HIV | 9 (0.8) | 0 (0.0) | 9 (0.8) |  | 1.000 |  |  |  |  |
| Solid tumor | 88 (8.2) | 4 (5.0) | 92 (8.0) |  | 0.312 |  |  |  |  |
| Hematological malignancy | 25 (2.3) | 1 (1.3) | 26 (2.2) |  | 1.000 |  |  |  |  |
| Solid organ transplantation | 16 (1.5) | 4 (5.0) | 20 (1.7) |  | **0.044** |  |  |  |  |
| Cirrhosis | 10 (0.9) | 1 (1.3) | 11 (1.0) |  | 0.547 |  |  |  |  |
| Charlson Comorbidity Index | 3 (1-5) | 4 (2-6) | 3 (1-5) |  | **0.005** |  |  |  |  |
| **Vital signs at admission^+^** |  |  |  |  |  |  |  |  |  |
| Temperature (ºC) | 36.6 (35.9-37.1) | 36.4 (35.8-37.6) | 36.5 (35.9-36.9) |  | 0.852 |  |  |  |  |
| APACHE | 16.0 (12.0-22.5) | 15.0 (13.3-23.8) | 16.0 (13.0-23.3) |  | 0.424 |  |  |  |  |
| SOFA | 2.53 (1.0-3.0) | 2.73 (2.0-4) | 2.56 (1.0-3.25) |  | 0.297 |  |  |  |  |
| **Laboratory parameters at admission^+^** |  |  |  |  |  |  |  |  |  |
| Ferritin (ng/mL) | 458.0 (302.5-1132.5) | 402.0 (160.0-883.0) | 494.0 (243.0-1002.0) |  | **0.012** |  |  |  |  |
| Ferritin < 402 (ng/mL) | 323 (34.6) | 27 (50.9) | 350 (35.5) |  | **0.016** |  | 2.28 | 1.21 – 4.29 | **0.011** |
| Leukocyte count (×10^9^/L) | 6.7 (5.0-9.3) | 8.7 (6.3-13.00) | 6.6 (5.0-9.2) |  | **<0.001** |  |  |  |  |
| Leukocyte count >8.7×10^9^/L | 317 (29.7) | 39 (50.6) | 356 (31.1) |  | **<0.001** |  | 2.40 | 1.26 – 4.45 | **0.004** |
| Neutrophil count (×10^9^/L) | 5.1 (3.5-7.7) | 6.8 (4.8-11.1) | 4.9 (3.5-7.4) |  | **<0.001** |  |  |  |  |
| Lymphocyte count (×10^9^/L) | 0.8 (0.6-1.2) | 0.8 (0.5-1.15) | 0.9 (0.6-1.2) |  | 0.230 |  |  |  |  |
| Platelet count (×10^9^/L) | 195.0 (148.0 -256.0) | 234.0 (162.0 -301.5) | 199 (153.0 -255.0) |  | **0.019** |  |  |  |  |
| C-RP (mg/L) | 97.7 (51.8-167.0) | 101.20 (50.6-170.9) | 80.3 (33.8-142.5) |  | 0.755 |  |  |  |  |
| Fibrinogen (mg/dL)**^+^** | 756.0 (634.0-874.0) | 702.0 (600.0-836.0) | 720.5 (603.0-849.0) |  | 0.097 |  |  |  |  |
| Procalcitonin (ng/mL)**^+^** | 0.12 (0.06-0.34) | 0.2 (0.06-0.78) | 0.1 (0.05-0.27) |  | **0.010** |  |  |  |  |
| Lactate dehydrogenase (U/L)**^+^** | 299.0 (234.0-410.0) | 307.3 (257.4-523.3) | 273 (218.9-374.0) |  | 0.138 |  |  |  |  |
| IL-6 (pcg/mL) **^+^** | 47.8 (26.5-88.7) | 48.7 (20.5-152.3) | 39.8 (20.6-79.0) |  | 0.685 |  |  |  |  |
| D-dimer (ng/mL) **^+^** | 735.0 (466.0-1284.0) | 1017.0 (569.5-2169.5) | 762.0 (475.0 -1325.0) |  | **0.006** |  |  |  |  |
| INR**^+^** | 1.21 (1.13-1.34) | 1.21 (1.13-1.30) | 1.21 (1.13-1.36) |  | 0.835 |  |  |  |  |
| Creatinine Kinase (U/L) **^+^** | 93.0 (54.0-189.0) | 101.0 (59.0-211.5) | 86.0 (52.0-176.0) |  | 0.611 |  |  |  |  |
| **Hospital-acquired secondary infections*** |  |  |  |  |  |  |  |  |  |
| Bacteremia | 52 (4.8) | 6 (7.5) | 58 (5.0) |  | 0.291 |  |  |  |  |
| Respiratory bacterial infection | 121 (11.2) | 19 (23.8) | 140 (12.1) |  | **0.001** |  |  |  |  |
| Urinary tract infection | 114 (10.6) | 17 (21.3) | 131 (11.3) |  | **0.004** |  |  |  |  |
| *Clostridioides difficile* | 3 (0.3) | 1 (1.3) | 4 (0.3) |  | 0.153 |  |  |  |  |
| ESBL******** | 23 (12.6) | 4 (14.8) | 27 (12.9) |  | 0.753 |  |  |  |  |
| MRSA******** | 4 (3.8) | 1 (3.7) | 5 (3.8) |  | 0.975 |  |  |  |  |
| XDR *P. aeruginosa* ******** | 7 (3.0) | 1 (5) | 8 (3.3) |  | 0.645 |  |  |  |  |
| VRE******** | 3 (2.2) | 1 (5.6) | 4 (2.6) |  | 0.401 |  |  |  |  |
| All Multidrug-Resistant microorganisms******** | 35.0 (19.1) | 7 (25.9) | 42 (20.0) |  | 0.410 |  |  |  |  |
| **Outcomes** |  |  |  |  |  |  |  |  |  |
| Invasive mechanical ventilation* | 205 (19.0) | 23 (28.8) | 228 (19.7) |  | **0.035** |  |  |  |  |
| ECMO* | 78 (7.2) | 13 (16.3) | 91 (7.9) |  | **0.004** |  |  |  |  |
| Length of hospital stay (days) | 11 (6-18) | 14 (7.3-32) | 10 (6-17) |  | **0.003** |  |  |  |  |
| ICU admission | 264 (24.5) | 26 (32.5) | 290 (25.1) |  | 0.112 |  |  |  |  |
| 30-day readmissions | 45 (4.2) | 8 (10.0) | 53 (4.6) |  | **0.025** |  |  |  |  |
| Death | 198 (18.4) | 16 (20.0) | 214 (18.6) |  | 0.720 |  |  |  |  |

**^+^***Within the first 48 hours after admission;* ********After 48 hours of admission;* *********Percentage of total bacteremia, respiratory bacterial infection, and urinary tract infection; MRSA, Methicillin-Resistance Staphylococcus aureus; XDR P. aeruginosa, Extensively drug-resistant Pseudomonas aeruginosa; ESBL, Extended-spectrum β-lactamases; VRE, Vancomycin-Resistant Enterococci; ECMO, Extracorporeal Membrane Oxygenation*

**Supplementary table 2. Antibiotic use in the four study periods in hospitalized COVID-19 patients.**

|  | **Period 1 (*n* =819)**  March 2020 -  May 2020 | **Period 2 (*n* =723)**  June 2020 - December 9, 2020 | **Period 3 (*n* =476)**  December 10, 2020 - March 15, 2021 | **Period 4 (*n*=103)**  March 16, 2021 - May 1, 2021 | **Total (N=2121)** | ***p-value*** |
| --- | --- | --- | --- | --- | --- | --- |
|  |  |  |  |  |  |  |
| Antibiotic at admission | 660 (80.6) | 137 (18.9) | 81 (17.0) | 20 (19.4) | 898 (42.3) | **<0.001** |
| Antibiotic after 48h of admission | 515 (62.9) | 78 (10.8) | 44 (9.2) | 10 (9.7) | 647 (30.5) | **<0.001** |
| Antibiotic after 72h of admission | 314 (38.3) | 60 (8.3) | 28 (5.9) | 6 (5.8) | 408 (19.2) | **<0.001** |
| DOT/100-patients-day | 62.5 (33.33-100) | 34.78 (14.29-66.67) | 32.46 (16.85-56.92) | 33.33 (12.12-60) | 50 (25-92.31) | **<0.001** |

*DOT, Days of therapy*

**Supplementary table 3. Comparison of sociodemographic features, clinical and analytical characteristics, and outcomes in hospitalized COVID-19 patients admitted to intensive care units according to antibiotic use at hospital admission.**

|  | **Without antibiotic at admission (*n* = 150)** | **With antibiotic at admission**  **(*n* = 220)** | **Total (*N* = 370)** |  | | |
| --- | --- | --- | --- | --- | --- | --- |
|  |  |  |  |  | ***p-value*** |  |
| **Socio-demographic characteristics** |  |  |  |  |  |  |
| Age | 62.0 (50.0-69.0) | 60.0 (53.0-66.0) | 61.0 (52.0-68.0) |  | 0.314 |  |
| Male gender | 93 (62.0) | 171 (77.7) | 264 (71.4) |  | **0.001** |  |
| **Vital signs at admission^+^** |  |  |  |  |  |  |
| Respiratory bacterial co-infection | 6 (14.3) | 9 (11.8) | 15 (12.7) |  | 0.703 |  |
| Oxygen saturation <94 | 0 (0.0) | 12 (18.8) | 12 (17.9) |  | 1.000 |  |
| APACHE | 14 (12.00-19.25) | 17 (13.00-25.75) | 16 (13.00-23.25) |  | **0.024** |  |
| SOFA | 3.5 (3.00-3.75) | 3 (2-4) | 3 (2-4) |  | 0.589 |  |
| SIRS | 1 (0.7) | 34 (15.5) | 35 (9.5) |  | **<0.001** |  |
| **Laboratory parameters at admission^+^** |  |  |  |  |  |  |
| Ferritin (ng/mL) | 882 (485.75-1970.5) | 1125 (668.5-1773.5) | 1057 (603-1829) |  | 0.180 |  |
| Leukocyte count (×10^9^/L) | 7.8 (5.7-10.2) | 8.4 (5.5-12.5) | 8.1 (5.7-11.45) |  | 0.427 |  |
| Platelet count (×10^9^/L) | 219 (159-320) | 208 (155-275) | 215 (157.0-283.5) |  | 0.122 |  |
| C-RP (mg/L) | 97.7 (51.8-167.0) | 101.20 (50.6-170.9) | 80.3 (33.8-142.5) |  | 0.755 |  |
| Fibrinogen (mg/dL)**^+^** | 723 (604.75-850.75) | 722.5(603.5-722.5) | 810.5 (657.5-940.3) |  | **0.011** |  |
| Procalcitonin (ng/mL)**^+^** | 0.13 (0.06-0.38) | 0.30 (0.13-0.78) | 0.24 (0.09-0.64) |  | **<0.001** |  |
| Lactat dehydrogenase (U/L)**^+^** | 447.30 (338.48-566.73) | 426 (304.5-543.4) | 437 (312.7-553.8) |  | 0.487 |  |
| D-dimer (ng/mL) **^+^** | 862.0 (515.0-1725.0) | 885.0 (489.0-1489.0) | 876.0 (512.0-1595.0) |  | 0.918 |  |
| **Hospital-acquired secondary infections*** |  |  |  |  |  |  |
| Bacteremia | 25 (16.7) | 48 (21.8) | 73 (19.7) |  | 0.222 |  |
| Respiratory bacterial infection | 67 (44.7) | 93 (42.3) | 160 (43.2) |  | 0.648 |  |
| Urinary tract infection | 51 (34.0) | 65 (29.5) | 116 (31.4) |  | 0.365 |  |
| ESBL******** | 13 (8.6) | 21 (9.5) | 34 (9.1) |  | 0.667 |  |
| MRSA******** | 4 (2.6) | 2 (0.9) | 6.0 (1.6) |  | 0.192 |  |
| XDR *P. aeruginosa* ******** | 3 (2.0) | 5 (2.3) | 8.0 (2.1) |  | 0.813 |  |
| All Multidrug-Resistant microorganisms******** | 21 (14.0) | 26 (11.8) | 47.0 (12.7) |  | 0.616 |  |
| **Outcomes** |  |  |  |  |  |  |
| Length of hospital stay (days) | 22.0 (13.8-38.0) | 24 (14.0-43.8) | 23 (14.0-40.0) |  | 0.339 |  |
| 30-day readmissions | 5.0 (3.3) | 3 (1.4) | 8 (2.2) |  | 0.278 |  |
| Death | 42.0 (28.0) | 55 (25.0) | 97 (26.2) |  | 0.519 |  |
| **Antibiotic consume** |  |  |  |  |  |  |
| DOT/100-patients/day | 26.6 (12.2-39.7) | 38.3 (17.4-71.4) | 34.7 (16.4-68.6) |  | **0.005** |  |

**^+^***Within the first 48 hours after admission;* ********After 48 hours of admission;* *********Percentage of total bacteremia, respiratory bacterial infection, and urinary tract infection; MRSA, Methicillin-Resistance Staphylococcus aureus; XDR P. aeruginosa, Extensively drug-resistant Pseudomonas aeruginosa, Extended-spectrum β-lactamases; DOT, Days of Therapy*
